# Polypyrrole Porous Micro Humidity Sensor Integrated with a Ring Oscillator Circuit on Chip

**DOI:** 10.3390/s101110095

**Published:** 2010-11-10

**Authors:** Ming-Zhi Yang, Ching-Liang Dai, De-Hao Lu

**Affiliations:** Department of Mechanical Engineering, National Chung Hsing University, Taichung, 402, Taiwan; E-Mails: g9761312@mail.nchu.edu.tw (M.-Z.Y.); g9561109@mail.nchu.edu.tw (D.-H.L.)

**Keywords:** humidity sensors, polypyrrole, CMOS-MEMS, ring oscillator circuits

## Abstract

This study presents the design and fabrication of a capacitive micro humidity sensor integrated with a five-stage ring oscillator circuit on chip using the complimentary metal oxide semiconductor (CMOS) process. The area of the humidity sensor chip is about 1 mm^2^. The humidity sensor consists of a sensing capacitor and a sensing film. The sensing capacitor is constructed from spiral interdigital electrodes that can enhance the sensitivity of the sensor. The sensing film of the sensor is polypyrrole, which is prepared by the chemical polymerization method, and the film has a porous structure. The sensor needs a post-CMOS process to coat the sensing film. The post-CMOS process uses a wet etching to etch the sacrificial layers, and then the polypyrrole is coated on the sensing capacitor. The sensor generates a change in capacitance when the sensing film absorbs or desorbs vapor. The ring oscillator circuit converts the capacitance variation of the sensor into the oscillation frequency output. Experimental results show that the sensitivity of the humidity sensor is about 99 kHz/%RH at 25 °C.

## 1. Introduction

Humidity sensors that are used to measure and monitor environmental humidity are important devices in many industrial and biomedical fields. The micro electromechanical system (MEMS) technique has become popular for the miniaturization of sensors. Humidity sensors fabricated by the MEMS technique have the advantages of small size, high performance, easy mass-production and low cost [1]. Several studies have recently used the MEMS technique to manufacture micro humidity sensors. For instance, Lee *et al.* [2] presented a polyimide humidity sensor with a micro-bridge structure created using front-side etching with XeF_2_ gas. The humidity sensor was a capacitive type, and the polyimide was locally cured at a temperature over 350 °C for 1 hour by the MEMS micro hotplate. The humidity sensor exhibited a sensitivity of 0.77 pF/%RH and a hysteresis of 0.61 %RH. Miyoshi *et al.* [3] utilized the soft-MEMS technique to manufacture a flexible humidity sensor. The humidity sensor was a sandwich structure with a hydrophilic poly-tetrafluoroethylene membrane located between two gold deposited layers. A resistive humidity sensor proposed by Li *et al.* [4] was fabricated using the multi-user MEMS process. The sensing material of the humidity sensor was the organic conductive polymer—poly(3,4-ethylenedioxythiophene)—that was prepared by electrochemical deposition, and filled in a narrow air gap between two nickel electrodes. The resistance of the sensor changed from 37 to 62 Ω when the relative humidity varied from 22 to 99.9 %RH at room temperature. Juhász *et al.* [5] developed two capacitive humidity sensors using a semiconductor process and anodic oxidation. The sensing layer of the sensors, which was porous alumina, was produced by electrochemical oxidation of aluminum thin film under anodic bias. One sensor had a vapor-permeable palladium upper electrode, and the other had an electroplated gold-grid electrode. The highest sensitivity of the sensors was about 15 pF/%RH. Chen *et al.* [6] used the MEMS technique to construct a resistive humidity sensor, which its structure contained a freestanding cantilever. The cantilever was a composite structure comprising a layer of platinum deposited on a silicon nitride layer and then covered with a polyimide sensing layer. When the polyimide sensing layer absorbed vapor, the cantilever produced a deflection, leading to the platinum layer generated a change in resistance. These humidity sensors [2–6] did not have integrated circuits-on-a-chip, so they required coupling with circuits by packaging, resulting in an increase in package cost. Integrating humidity sensors with circuits on chip helps to enhance the performance and reduce the packaging cost. Therefore, in this work a humidity sensor integrated with a ring oscillator circuit on chip is developed.

In this study, we investigate the fabrication of a micro humidity sensor integrated with a ring oscillator circuit on chip using the CMOS-MEMS technique. The sensing material of the sensor is polypyrrole. The post-process uses wet etching to etch the sacrificial layers and the polypyrrole is coated on the humidity sensor. The sensor produces a change in capacitance when the sensing film absorbs or desorbs vapor. The ring oscillator circuit converts the capacitance of the humidity sensor into the oscillation frequency output.

## 2. Design of the Humidity Sensor

**Figure 1.**
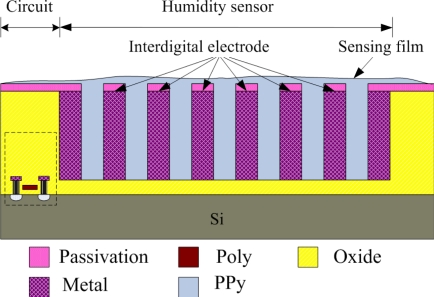
Cross-sectional view of the integrated humidity sensor.

Figure 1 illustrates the cross-sectional view of the integrated humidity sensor, where the thickness and width of the interdigital electrodes are about 8 μm and 5 μm, respectively, and the gap between the electrodes is 4 μm. The integrated humidity sensor is composed of a sensing film, a sensing capacitor and a ring oscillator circuit. The humidity sensor is a capacitive type. The sensing film of the humidity sensor is polypyrrole that is synthesized by the chemical polymerization method. The polypyrrole film is coated on the interdigital electrodes. The capacitance variation of the sensing capacitor depends on the dielectric constant of the sensing film. The sensing capacitor changes in capacitance when the polypyrrole film absorbs or desorbs vapor, leading to the capacitance of the humidity sensor changes.

**Figure 2.**
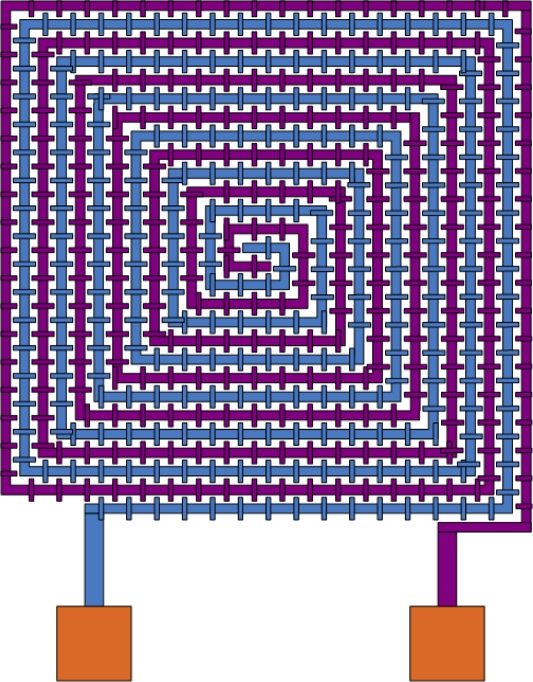
Schematic structure of the sensing capacitor.

Figure 2 shows the structure of the sensing capacitor that is constructed of spiral interdigital electrodes [7]. The spiral interdigital electrodes can increase the capacitance of the sensor, resulting in enhancing the sensitivity of the sensor. The material of spiral interdigital electrodes is the metal layers of the CMOS process.

**Figure 3.**
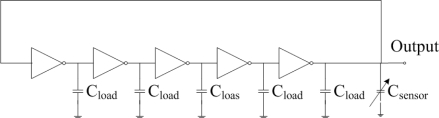
five-stage ring oscillator circuit for the humidity sensor.

A five-stage ring oscillator is adopted as the sensing circuit owing to the fact its phase noise is smaller than that of three-stage ring oscillator, and it has a stable output signal [8]. Figure 3 illustrates the ring oscillator circuit, where C_sensor_ represents the capacitance of the humidity sensor and C_load_ is the load capacitance. The ring oscillator circuit is used to convert the capacitance variation of the sensing capacitor into the frequency output. As shown in Figure 3, the circuit is a five-stage ring oscillator, which is connected by five identical inverters. The five inverters form a voltage feedback loop.

The ring oscillator circuit connects with odd inverters does not have a stable operating point. Therefore, the circuit will oscillate when any of the odd inverter input or output voltage separates from the unstable operating point. The oscillation frequency f_sensor_ of the ring oscillator circuit is given by [9]

(1) $$f_{\mathrm{sensor}}=\frac{1}{10\tau_{\mathrm{inv}}+2\tau_{\mathrm{sensor}}}=\frac{1}{\frac{10C_{\mathrm{load}}\Delta V}{I_{\mathrm{ave}}}+\frac{2C_{\mathrm{sensor}}\Delta V}{I_{\mathrm{ave}}}}$$

where τ_inv_ is the delay time associated with the inverters, τ_sensor_ the delay time associated with the humidity sensor, C_load_ the load capacitance, C_sensor_ the humidity sensor capacitance, and ΔV and I_ave_ are the threshold voltage and average current, respectively. According to Equation (1), we know that the oscillation frequency of the ring oscillator circuit changes while the capacitance of the humidity sensor varies.

**Figure 4.**
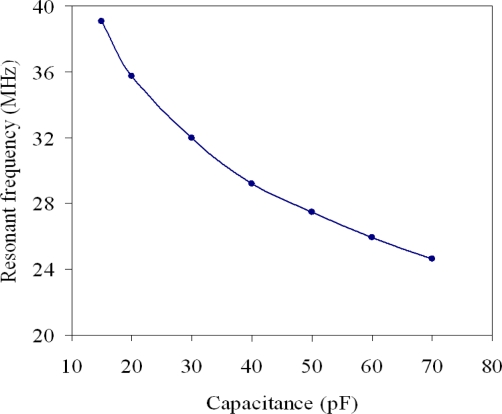
Simulated results of the humidity sensor.

The professional circuit simulation software, HSPICE, was used to analyze the characteristics of the ring oscillator circuit. Figure 4 presents the simulated results of the five-stage ring oscillator circuit. In the simulation, the load capacitance is 2 pF, and the humidity sensor capacitance varies from 15 to 70 pF. The oscillation frequency of the ring oscillator circuit varies from 39.2 to 24.6 MHz as the capacitance of the humidity sensor rises from 15 to 70 pF.

## 3. Fabrication of the Humidity Sensor

**Figure 5.**
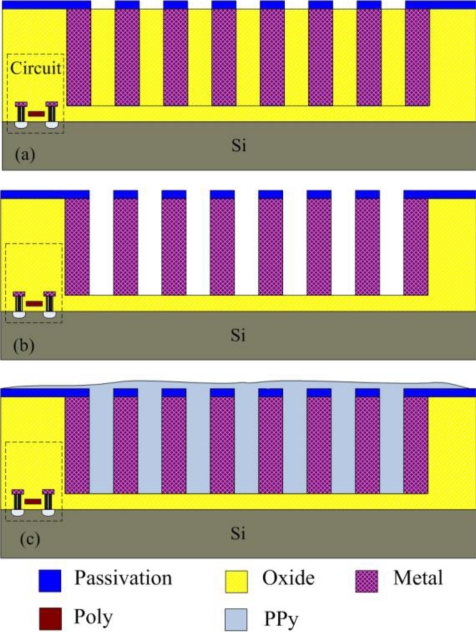
Fabrication process of the humidity sensor: **(a)** after the CMOS process, **(b)** etching the sacrificial layer, **(c)** coating the sensing film.

The commercial 0.35 μm CMOS process of Taiwan Semiconductor Manufacturing Company (TSMC) was utilized to manufacture the integrated humidity sensor chip. The chip requires a post-process to etch the sacrificial layers and to coat the polypyrrole film after completion of the CMOS process. Figure 5 illustrates the process flow of the integrated humidity sensor chip.

Figure 5(a) presents the integrated humidity sensor after completion of the CMOS process. In the humidity sensor, the silicon dioxides are adopted as the sacrificial layer, and the metal layers are used as the sensing capacitor. The sacrificial layer must be removed. Figure 5(b) shows that the chip is immersed in Silox to etch the silicon dioxide, and to obtain the gaps of interdigital electrodes in the sensing capacitor.

**Figure 6.**
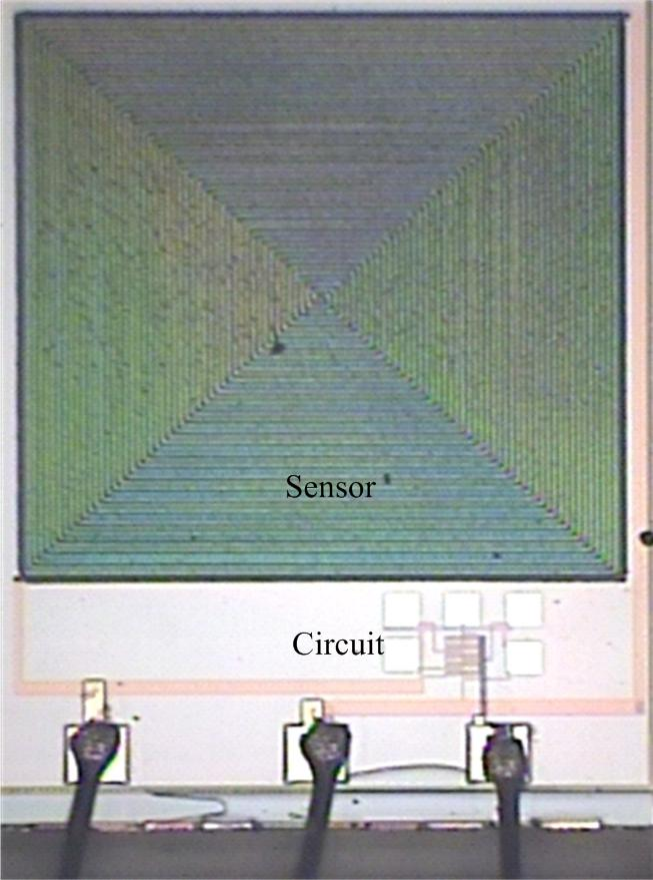
Photograph of the integrated humidity sensor after the wet etching process.

**Figure 7.**
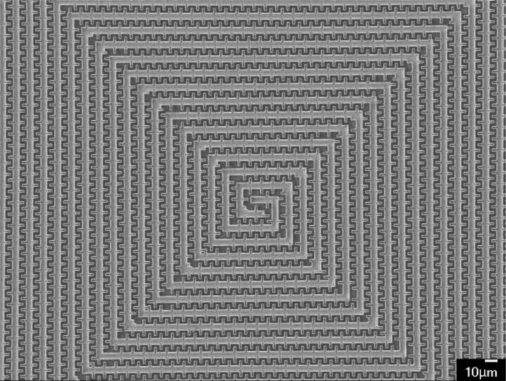
SEM image of the sensing capacitor.

Figure 6 displays a photograph of the humidity sensor chip after the wet etching process and wire bonding process. Figure 7 shows a scanning electron microscopy (SEM) image of the spiral interdigital electrode sensing capacitor. Finally, the polypyrrole is coated on the sensing capacitor as shown in Figure 5(c). The polypyrrole, was synthesized by the chemical polymerization method [10,11] according to the following steps: (1) 5 mL pyrrole (C_4_H_5_N) is mixed with 50 mL deionized water and stirred continuously; (2) 10 g anhydrous ferric chloride (FeCl_3_) is mixed with 30 mL deionized water and stirred continuously; (3) the solution of FeCl_3_ is added into the pyrrole solution with stirring for 48 h. The resulting product is filtered, dropped on the humidity sensor chip. Then, the chip is followed by calcination in air at 120 °C for 3 h.

**Figure 8.**
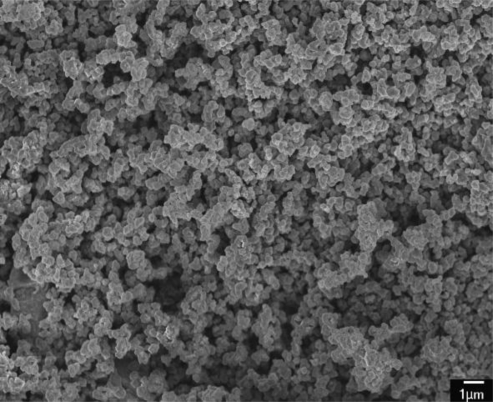
SEM image of the polypyrrole film

Figure 8 demonstrates a SEM image of the polypyrrole film that displays nanoparticle and porous structures. The nanostructures help to enhance the humidity sensitivity of the film. The diameter of grains in the polypyrrole film is about 0.3–0.5 μm, and the gap between the electrodes is 4 μm. Therefore, the polypyrrole film can fill into the gap between the electrodes.

## 4. Results and Discussion

**Figure 9.**
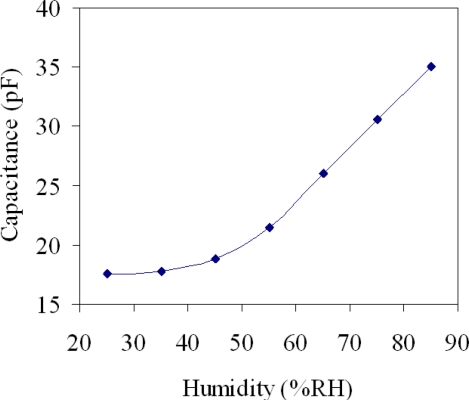
Measured capacitance in the humidity sensor.

The performance of the humidity sensor was measured by a power supply, a LCR meter, a spectrum analyzer and a test chamber (GTH-099-40-1P, Giant Force Instruments Enterprise Co.). The test chamber could supply a temperature range of 0–100 °C and a humidity range of 25–95 %RH. Temperature and humidity in the test chamber can be tuned separately and kept at constant levels. The spectrum analyzer was utilized to record the signal response of the sensor to humidity changes. The LCR meter was adopted to detect the capacitance of the sensor. The humidity sensor was set in the test chamber. The power supply provided a bias voltage of 3.3 V to the sensor. In order to characterize the capacitance variation of the sensor to humidity, the sensor without the ring oscillator circuit was tested. The test chamber supplied different humidity to the humidity sensor. When the humidity rose or dropped, the humidity sensor changed in capacitance. The capacitance variation of the humidity sensor was measured by the LCR meter. Figure 9 shows the measured capacitance in the humidity sensor. In this investigation, the temperature was maintained constant at 25 °C and the humidity was increased from 25 %RH to 85 %RH in 35 min. The results revealed that the capacitance of the sensor changed from 17.5 to 35.1 pF as the humidity varied from 25 to 85 %RH. The humidity sensor with the ring oscillator circuit was tested. The capacitance of the humidity sensor was converted into the oscillation frequency using the ring oscillator circuit. The test chamber provided different humidity to the humidity sensor. The spectrum analyzer was employed to measure the output frequency of the ring oscillator circuit.

**Figure 10.**
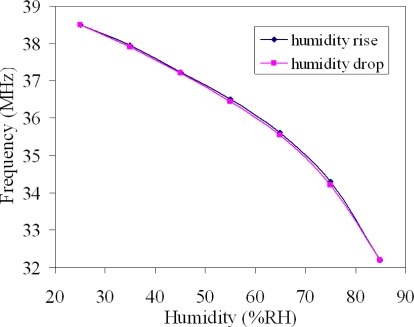
Humidity hysteresis curve of the humidity sensor at 25 °C.

Figure 10 displays the output frequency of the humidity sensor. In this measurement, the humidity was increased from 25 %RH to 85 %RH in 35 min and then dehumidified to 25 %RH at the same rate, and the temperature was kept constant at 25 °C. The experimental results depicted that the humidity sensor had a small humidity hysteresis.

**Figure 11.**
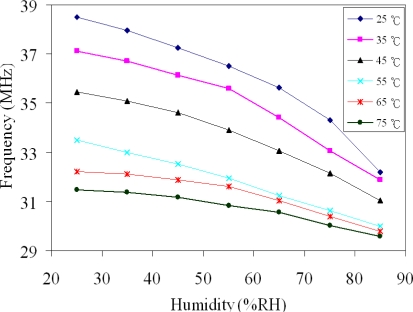
Measured results of the humidity sensor at different temperatures.

In order to understand the influence of temperature, the output frequency of the humidity sensor was tested at different temperatures. Figure 11 shows the measured output frequency of the humidity sensor at different temperatures. The sensitivity of the sensor, which was obtained by the linear fitting to the data in Figure 11, was 99 kHz/%RH at 25 °C, 59 kHz/%RH at 55 °C and 32 kHz/%RH at 75 °C. Thereby, the sensor had a better sensitivity at room temperature.

**Figure 12.**
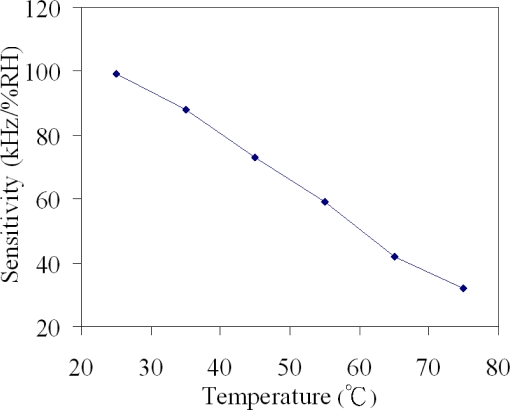
Relation between humidity sensitivity and temperature.

Figure 12 presents the relation between the humidity sensitivity and temperature in accordance with the above results. The fitted curve equation of the data (Figure 12) was given by:

(2) S=−1.39T+135

where *S* represents the sensitivity of the humidity sensor and *T* is the temperature. According to Equation (2), we knew that the sensitivity of the sensor decreased as the temperature rose.

Dai [12] proposed a capacitive humidity sensor with circuit fabricated using the CMOS-MEMS technique. The sensing material of the sensor was polyimide, and the circuit was a three-stage ring oscillator. The humidity sensor had a sensitivity of 14.5 kHz/% RH at 25 °C. Comparing with Dai [12], the sensitivity of the sensor in this work exceeded that of Dai [12]. Beside, the experimental results showed that the ring oscillator circuit was normally operation after the wet etching post-process, indicating that the post-process was compatible with the commercial CMOS process.

## 5. Conclusions

This humidity sensor integrated with a five-stage ring oscillator circuit has been implemented using the commercial 0.35 μm CMOS process and the post-process. The humidity sensor was composed of a sensing film and a sensing capacitor. The sensing film was polypyrrole, that was synthesized by the chemical polymerization method. The electrodes of the sensing capacitor were designed with a spiral interdigital shape that could increase the sensor capacitance. The post-process used wet etching to etch the sacrificial layers, and the polypyrrole was coated on the sensing capacitor. The ring oscillator circuit converted the capacitance variation of the sensor into the frequency output. The experiments showed that the ring oscillator circuit was normally operation after the wet etching post-process. Thereby, the post-process was compatible with the commercial CMOS process. The measured results depicted that the humidity sensor had a sensitivity of 99 kHz/%RH at 25 °C, and its sensitivity exceeded that of Dai [12]. The humidity sensor had the advantages of small area and high sensitivity.
